# Supplementary material for: Hsp70 Peptides Induce TREM-1-Dependent and TREM-1-Independent Activation of Cytotoxic Lymphocytes
Source: Int J Mol Sci. 2025 Oct 7;26(19):9750. doi: 10.3390/ijms26199750 (PMC12525431; doi:10.3390/ijms26199750)
Supplement: Supplementary file 1 [file ijms-26-09750-s001.zip › Supplemental Figures S1-S8.pdf]

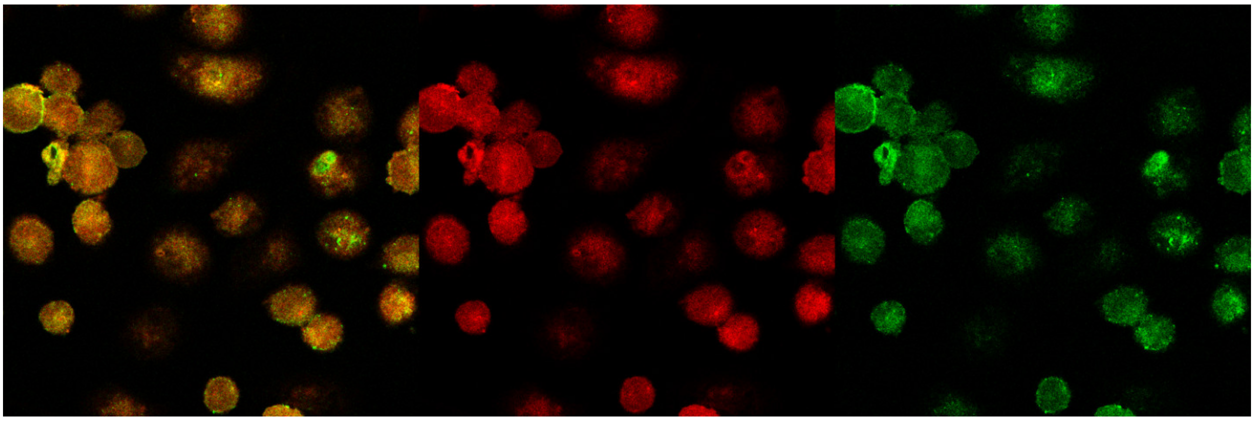

**Supplemental Figure S1.** Typical confocal photos of TREM-1 (green) and N7 (red) and layers superposition on the surface of THP-1 cells.

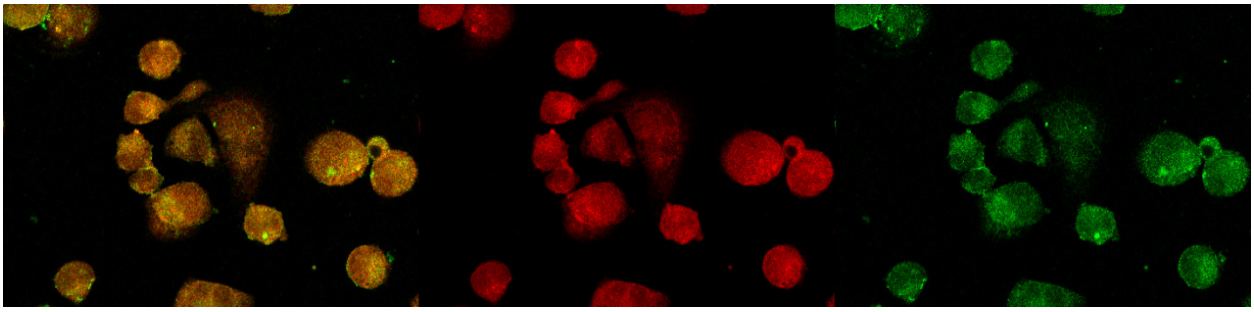

**Supplemental Figure S2.**Typical confocal photos of TREM-1 ( green) and N7 (red) and layers superposition on the surface of THP-1 cells.

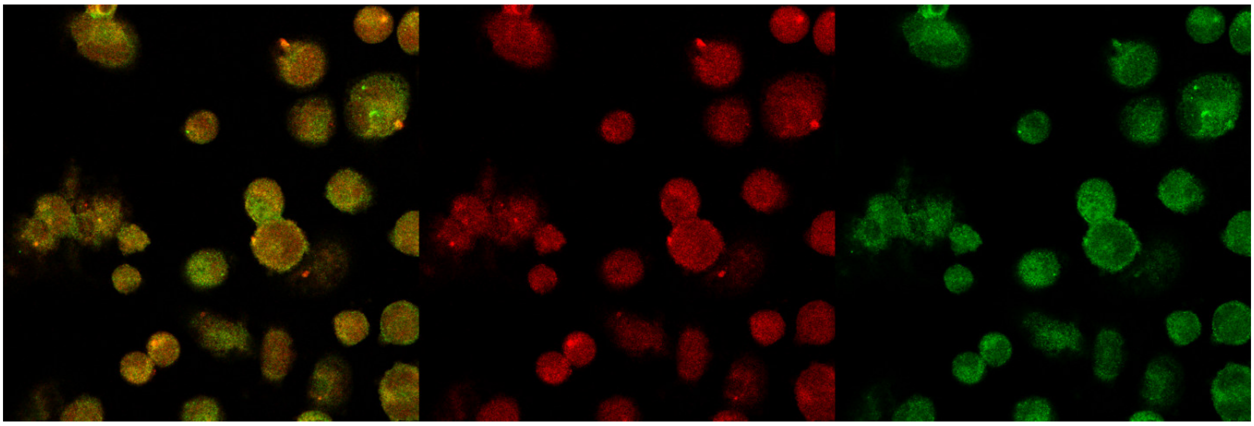

**Supplemental Figure S3.** Typical confocal photos of TREM-1 ( green) and N7 (red) and layers superposition on the surface of THP-1 cells.

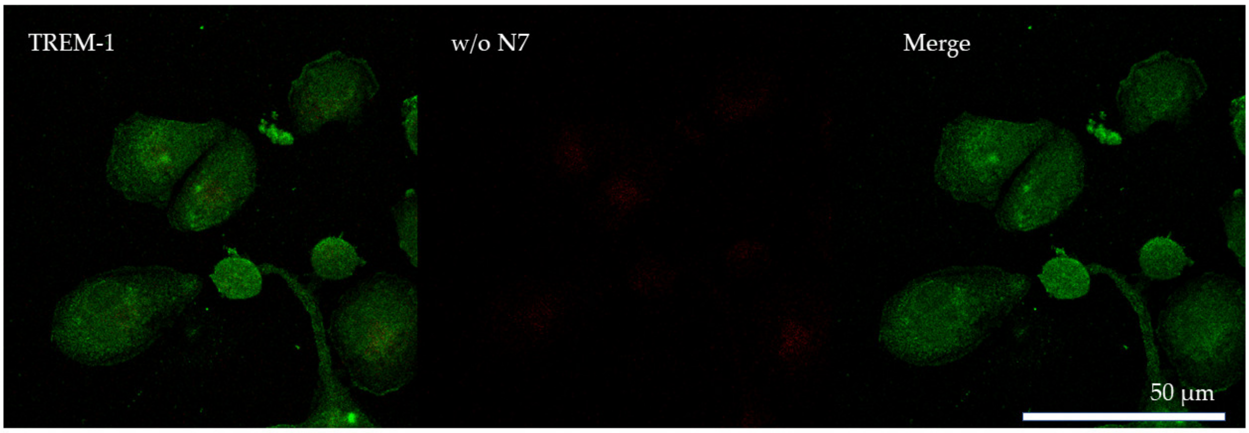


**Supplemental Figure S4.** Confocal photo of TREM-1 ( green) and ab Hsp70 (red) and layers superposition on the surface of THP-1 cells without addition of N7 peptide.

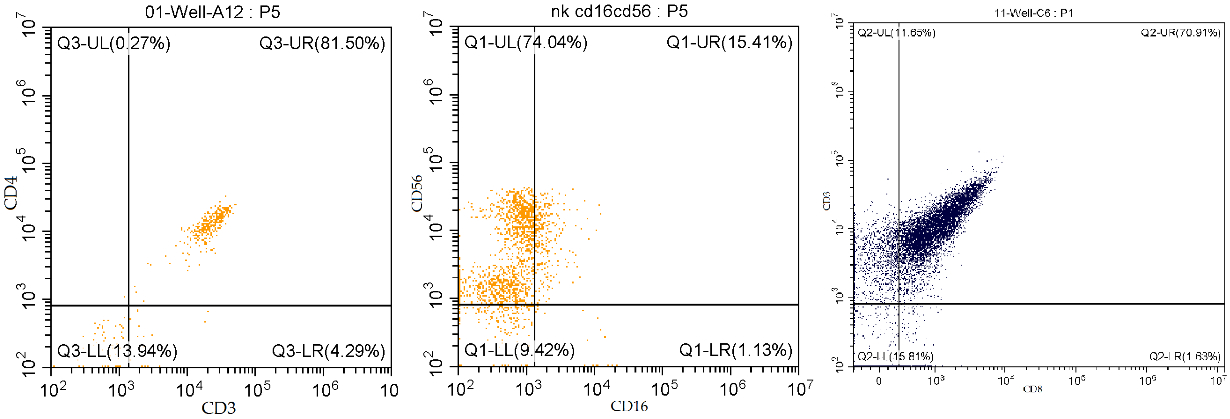


**Supplemental Figure S5.** Typical flow cytometry analysis of the PBMC subpopulations (CD4 T cells, NK cells and CD8 T cells) after magnetic separation before adding to K562 cells.

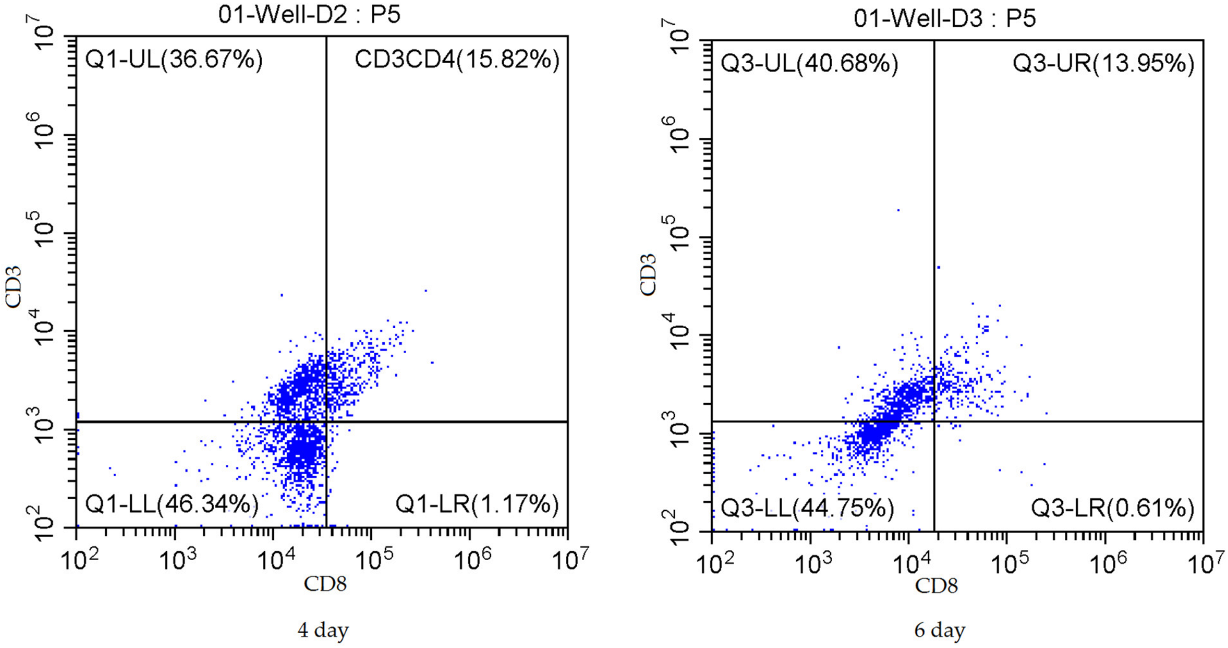


**Supplemental Figure S6.** Typical flow cytometry analysis of the PBMCs lymphocytes on day 4 and 6.

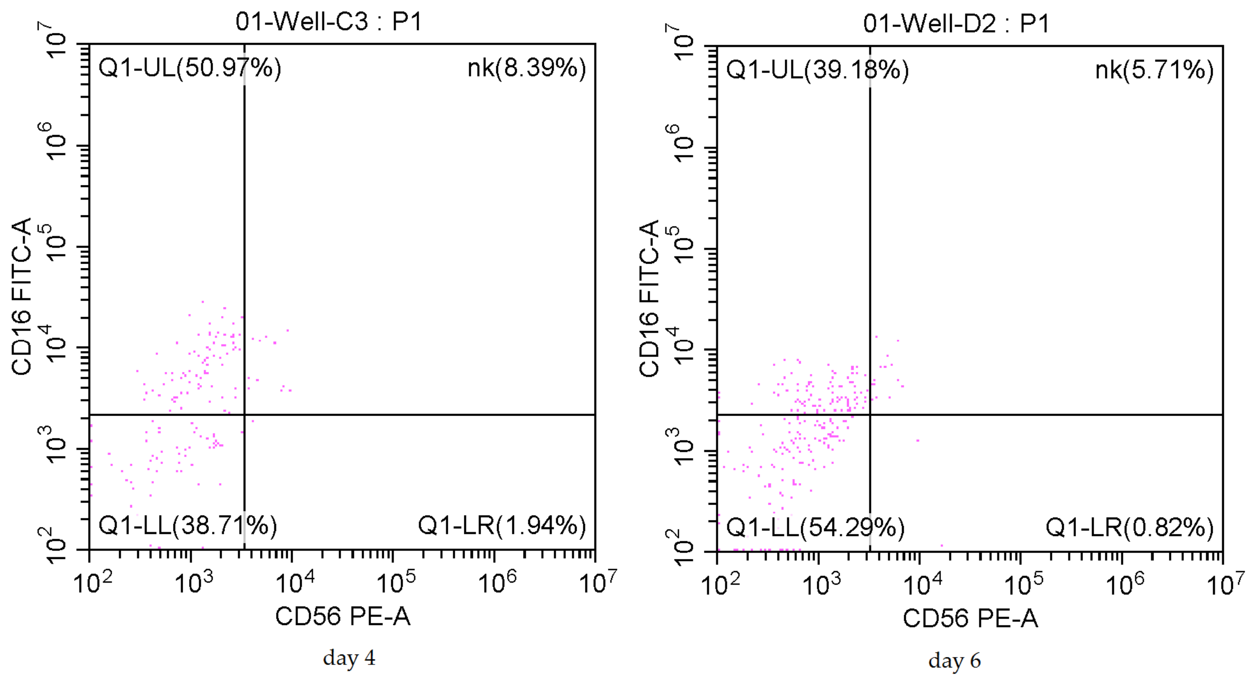


**Supplemental Figure S7.** Typical flow cytometry analysis of the PBMCs NK cells on day 4 and 6.

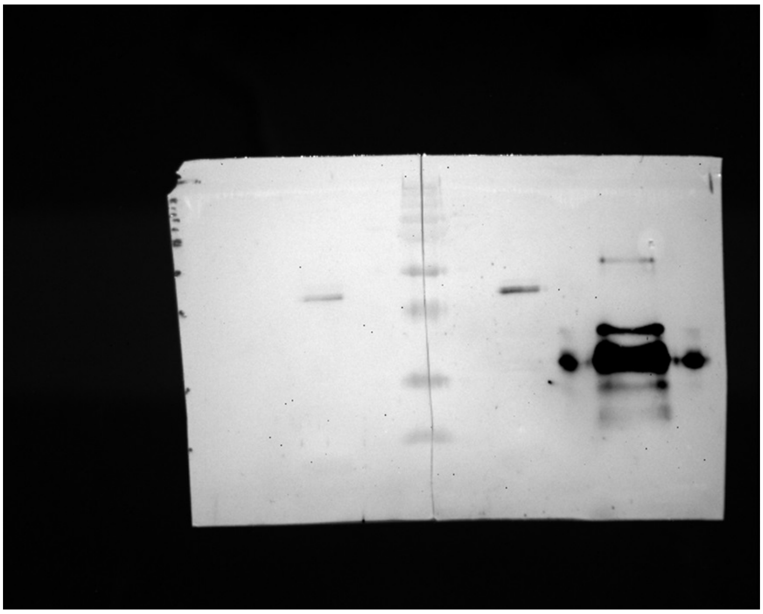

**Supplemental Figure S8.** Original gel of figure 6.
